# Leader’s Perception of Corporate Social Responsibility and Team Members’ Psychological Well-Being: Mediating Effects of Value Congruence Climate and Pro-Social Behavior

**DOI:** 10.3390/ijerph19063607

**Published:** 2022-03-18

**Authors:** Jae-Geum Jeong, Suk Bong Choi, Seung-Wan Kang

**Affiliations:** 1 College of Global Business, Korea University, Sejong City 30019, Korea; j2gok@korea.ac.kr; 2 College of Business, Gachon University, Seongnam 13120, Korea

**Keywords:** corporate social responsibility, value congruence climate, pro-social behavior, psychological well-being, multi-level analysis

## Abstract

Previous research, that showed that corporate social responsibility (CSR) had positive effects on the corporate image and performance, has attracted much attention and resulted in an increasing number of follow-up studies. However, CSR-related activities are focused on their effect on external stakeholders, although they are social service activities geared towards internal and external stakeholders, thus showing a research gap regarding the effects of internal stakeholders on organizational effectiveness. Therefore, this study investigated the mediating effects of the value congruence climate and prosocial behavior among the team members in the relationship between leader’s CSR perception and team members’ psychological well-being, using a multilevel analysis of the relationship between the team and individual level factors. For the empirical analysis, 69 teams (334 employees) were sampled from 23 Korean small and medium-sized enterprises (SMEs). Analyses revealed a positive effect of a leader’s CSR perception on the team members’ psychological well-being. Furthermore, a leader’s CSR perception had a positive effect on his/her team’s value congruence environment and team members’ prosocial behavior. The team’s value congruence environment and team members’ prosocial behavior were found to mediate the relationship between the leader’s CSR perception and team members’ psychological well-being. The relationships among these variables were investigated using a multilevel analysis model capable of simultaneous validation of team- and individual-level factors associated with team members’ psychological well-being. Future research directions were then discussed based on the theoretical and practical implications and limitations of the study results.

## 1. Introduction

Corporate social responsibility (CSR) activities were initiated by Howard [1] who defined CSR as the pursuit of policies desirable in terms of the societal objectives and values and laid out social obligations of companies as business ethics [2]. The CSR activities can be defined as economic, legal, ethical, and philanthropic duties of firms to pursue interests through honest and ethical business operations and provide services to improve community well-being [3]. Maignan and Ferrell [3,4] divided CSR activities into economic, legal, ethical, and philanthropic responsibilities. The CSR activities serve as an important means of eliciting social consensus going beyond profit-making, which is the ultimate goal of all firms [4]. They also constitute underlying force for sustained competitive advantage by establishing mutually preferential relationships with various stakeholders and diffusing positive corporate images [5]. Not only do CSR activities play a strategic marketing role of a company [6], but they also serve as a means of enhancing corporate solidarity by broadening mutual understanding among the organizational members and providing motivational support [7,8,9].

However, some researchers have put forward critical interpretations; for example, CSR activities cannot provide a normative reference for social responsibilities in actual business practices [10], and they are only a surreptitious expression of corporate power [11]. Despite such critical views, reports on the social and international demands for and positive effects of CSR activities have attracted increasing interest with positive corporate outcome expectations. From the marketing perspective, there are studies on quantifying marketing potential or profits following CSR activities [6,12], investigating consumer responses [13,14], dealing with positive effects of charity activities [15], and reporting environmental conservation activities [16,17]. However, the primary concern of these studies is the relationships with external stakeholders at the corporate level, and only few studies have dealt with relationships with internal stakeholders [18,19,20].

The stakeholder theory views companies as the links to various stakeholders [21], where stakeholders can be categorized into external stakeholders (e.g., consumers, suppliers, competitors, communities, media, nation, and government) and internal stakeholders (e.g., employees, managers, investors, and board of directors) [2]. Of particular interest, internal stakeholders are organizational members who determine and implement policies regarding CSR activities. However, employees rarely have direct interactions with the top management and are often led to judge through external activity indicators instead of acquiring concrete information about CSR activities through internal communication [20]. Employees of a company will judge whether and how their company deals with the internal and external environments and local community through its CSR activities [18] and build trust that it will treat them and their organization in the same manner. Such trust expands their interest in and positive perception of CSR activities and enhance organization-based self-esteem and strength of sense of identification with the organization [22,23,24]. This suggests that the CSR-based corporate image and positive perception by internal stakeholders are equality important.

On the other hand, given that CSR activities are planned and conducted at the corporate level in pursuit of continuous growth and sustainable competitive advantage, human resources such as team-level organizations within a company can be a vital source of competitive advantage [25,26]. In a team-level corporate unit, interactions with the team leader and team members are of crucial importance. The leader–member exchange (LMX) theory posits that a good leader–member relationship intensifies mutual trust and has a positive effect on job attitude [27,28] and affective organizational commitment [29,30,31]. These results highlight the importance of the leader’s role within a team. Thus, the leader’s perception of the CSR activities and related information will likely be shared through his/her interactions with the team members. These discussions demonstrate the importance of CSR activities for the relationship among the internal stakeholders, especially the leader–members relationship. However, previous studies on CSR activities have three limitations. First, they revolve around the aspect of marketing (consumer), which represents relationships with external stakeholders, or ethical and charity activities for the local community (e.g., donation and environmental conservation) [6,14,15,16]. Second, although CSR activities are closely associated with internal as well as external stakeholders, CSR-related internal stakeholders have been examined only to a limited extent such as individual level analysis. Third, given the potential of CSR activities for creating a corporate environment, the leader’s CSR perception can have a great effect on the team members behavior and effectiveness [5]. Studies on the inherent attributes, such as leader’s perception of CSR activities and team environment, have been conducted at individual level despite the necessity of examining them at the multi-level [32]. To bridge this research gap, this study aims to identify the comprehensive effect of relationship among team leader’s perception of CSR, the value congruence environment of the team, and the prosocial behaviors of the team members by using multi-level analysis model. This study seeks to broaden the understanding of CSR activities by examining the effect of such relationships on the team members’ psychological well-being [33,34,35].

The objectives of this study can be summarized as investigating three main relationships: effects of the leader’s perception of CSR on the team’s value congruence environment and team members’ prosocial behaviors; positive effects of the team’s value congruence environment and team members’ prosocial behaviors on the team members’ psychological well-being; the mediating effect of the team’s value congruence environment and team members’ prosocial behaviors on the relationship between leader’s perception of CSR and team members’ psychological well-being. By pursuing these objectives, this study is expected to contribute to an in-depth understanding of the cause-effect relationships regarding internal stakeholders that are neglected in the current research on CSR activities.

## 2. Theoretical Background and Hypotheses

### 2.1. Team Leader’s CSR Perception, Team’s Value Congruence Environment, and Team Members’ Pro-Social Behaviors

A team leader’s perception of CSR can be defined as the extent to which the team leader is aware of and agree with the company’s assumption of economic, legal, ethical, and philanthropic responsibilities to pursue interests through honest and upright business operations and provide services to contribute to the local community [3]. Maignan et al. [3] noted that employees of a socially active organization tend to feel committed to their employer, supporting his/her goals, and enjoy working in a company that promotes workplace experiences and minimizes profits. If a company strives to implement CSR activities, employee turnover is reduced [36,37,38] and employees’ motivation is increased [39]. It is also an efficient method to broaden the understanding of the organization and enhance solidarity among the employees [7,8,9]. These positive effects presuppose a sufficient perception of the company’s CSR activities by its employees. However, because team members have restricted access to the relevant information, unlike the team leader, they may be less aware of the company’s CSR activities. That is, in addition to the team leader’s sufficient perception of CSR, smooth interactions with team members are presupposed for the information related to the company’s CSR activities to be adequately provided to team members. Smooth interactions with team members are essential for broadening the understanding of their company’s CSR activities and diffusing their positive perception of their company. This can lead to the diffusion of the corporate ethical environment associated with its CSR activities among the team members, which in turn entails various positive results. According to Mayer et al. [12], the perception of the corporate ethical environment enhances job satisfaction and organizational commitment at the individual level, thus exerting positive effects on efficiency, social responsibility, organizational learning, and performance at the organizational level. Such an ethical environment can be built through the perception of and assimilation with the company’s CSR activities among the employees. As a result, employees become aware of the integrity of their company and get motivated to put more trust in the organization. This corporate environment automatically entails the team environment and can serve as an important path for boosting the team’s value congruence environment.

The value congruence environment of a team is the extent to which the belief determinant of individual attitude and behaviors is shared and encouraged among the team members [40,41]. Value congruence between team leader and team members suggests a high consensus between individual and organizational identities. The issue of value congruence has been intensively dealt with in research on the individual–organization fit, which means the degree to which an individual’s values match the organizational values and culture [42,43]. Individual and organizational value congruence is a very important unifying factor that steers the members of an organization towards one direction and enhances the overall performance of the organization. Value congruence means that the members of an organization do not merely agree to the values of the organization, but also strive to maintain, cherish, and adhere to the values [44]. Vveinhardt and Gulbovaitė [45] performed empirical analysis and developed a model for intensifying value congruence and assessed individual and organizational value congruence in seven different aspects (physical, occupational, social, moral, spiritual, economic, and aesthetic). Such a classification can serve as assessment criteria for building universal values in a society or organization and contribute to improving value congruence in the leader–member interaction based on the leader’s perception of CSR because such aspects coincide with the CSR-related legal and ethical activities or social support. In this situation, team members will experience more satisfaction with and commitment to their roles [46], which in turn will likely enhance the team and individual value congruence. A leader with a high perception of CSR activities can easily kindle the team members’ positive views and values regarding CSR activities, thus boosting the team’s value congruence environment. Furthermore, such a leader can inspire each team member into value congruence and the teams value congruence environment transferred through his/her attitude and behaviors in the interactions with the team members, thus reflecting the desirable positive sentiment towards the company’s CSR activities and higher level of overall value congruence.

Prosocial behaviors are generally defined as all voluntary acts that are beneficial to society and the organization to which one belongs [47,48]. More specifically, they are voluntary actions undertaken without expectations of rewards, such as assisting a colleague with task performance and taking on additional tasks going beyond the assigned roles and obligations [49,50]. Brief and Motowidlo [48] described prosocial behaviors as acts such as helping, sharing, donating, cooperating, and volunteering. Batson [51] argued that prosocial behaviors are acts aiming at reducing another’s distress and increasing another’s pleasure or happiness. Darley and Latane [52] noted that helping in an emergency occurs by recognizing that something is wrong, feeling the need to provide help after interpreting the event as an emergency, and helping. Such an act of helping can take place when helping coworkers during a team operation, whereby such prosocial behaviors are proportional to the level of sympathy [53]. In this context, it can be assumed that the team leader’s perception of CSR activities will have a positive effect on the team members, building positive affect among them and thus increasing their willingness toward prosocial behaviors. O’Reilly and Chatman [54] reported that CSR activities tend to reduce employee turnover and elicit behaviors of taking on additional roles. Mayer et al. [12] noted that perception of ethical climate increases job satisfaction, organizational commitment, and employees’ ethical behavior at the individual level, and have positive effects on efficiency, social responsibility, organizational learning, and performance at the organizational level. A devoted employee likes the organization, sees its future, and considers his/her willingness to continue personal devotion. A company engaged in active CSR takes a great interest in workplace environment and staff welfare, and its employees perform their own and additional roles with high self-esteem [55]. The stronger the humanistic orientation of a company, the more active its CSR activities, resulting in eliciting stronger devotions from its employees, and its corporate citizenship becomes more visible, resulting in more positive organizational effect [3]. Based on the above discussions, we assumed that the leader’s perception of CSR has a positive effect on the team’s value congruence and the team members’ pro-social behaviors. Thus, we hypothesized:

**Hypothesis** **1a.** *Leader’s perception of CSR will have a positive effect on the team’s value congruence environment.*

**Hypothesis** **1b.** *Leader’s perception of CSR will have a positive effect on the team members’ prosocial behaviors.*

### 2.2. Team’s Value Congruence Environment and Team Members’ Prosocial Behaviors and Psychological Well-Being

Well-being is a construct originating from positive psychology, which focuses on individual happiness [56], whereby happiness has evolved into the concept of well-being in positive organizational scholarship [57]. Well-being differentiates between subjective well-being and psychological well-being; whereas the former appears in the form of maximizing happiness by pursuing personal safety and pleasure [58], the former is explained with focus on mental health, abundance, and optimal psychological functioning [59]. Ryff and Keyes [60] proposed a six-factor model of psychological well-being (self-acceptance, positive relations, autonomy, environmental mastery, purpose in life, and a sense of personal growth): (1) Self-acceptance is a positive attitudes towards oneself, acknowledging and accepting multiple aspects of life and being satisfied with the past and current lives; (2) Positive relations can be built through affection, interest, familiarity, trust, and concerns about the welfare of others; (3) Autonomy refers to the capacity to be self-determining, independent, thinking and acting on one’s own accord, and self-controlling; (4) Environmental mastery is the capacity to manage the environment through familiarity with the surroundings, make effective use of surrounding opportunities, and create suitable contexts; (5) Purpose in life is having goals and a sense of life and giving meaning to the present and past life; (6) Personal growth represents a feeling of continued development, growth and expansion of oneself, openness to new experiences, recognition of one’s own potential, and expectation of improvement over time. To sum up, psychological well-being can be defined as a state of positive assessment of the meaning and goals of one’s past and present lives and anticipated future life satisfaction and happiness [60]. Conclusively, psychological well-being has diverse and complex meanings as shown by the model developed by Ryff and Keyes [60] and Jeong, Kang, and Choi [61].

We propose that psychological well-being is influenced by the team’s value congruence environment and the team members’ prosocial behaviors. Value congruence, which is a measure of match between individual and organizational values or cultures [43], reduces conflict factors within a team and makes team members become aware of the importance of their roles and more committed to the organization [46]. In this process, improvement of self-acceptance and positive relations can be expected to have a positive effect on psychological well-being through the mediation of the enhanced perception of one’s own role and establishment of mutually preferential relationship. Furthermore, value congruence is considered an important motivational factor for organizational identification [62]. Such organizational identification was described as a process of cognitive connection of the perception of organization to general, personal definition [63] and a cognitive and psychological isomorphism between the individual and organizational identities [64]. Such isomorphism can be interpreted in the sense of fusing the organizational identity into the life goal of team members and thus expanding the self-identity, which is expected to have a positive effect on their psychological well-being. Team members are led to evaluate one another in interactions and exchanges, whereby a good value congruence environment elicits a feeling of homogeneity due to a high value congruence. The seduction theory posits that the higher the mutual similarity, the higher the sympathy and the more positive the perception of each other [65], which can work as social and occupational attractiveness. To sum up, as the team environment enhances the level of value congruence and develops into cooperative relationship, team members’ individual level of satisfaction with work and collegial relationship will rise. Therefore, this study proposes that the team’s value congruence environment will be positively associated with positive relations with others, self-acceptance, and personal growth mentioned in relation to individual psychological well-being.

**Hypothesis** **2a.** *Team’s value congruence environment will have a positive effect on team members’ psychological well-being.*

Prosocial behavior is a concept covering a broad range of organizational citizenship behaviors going beyond the assigned roles. Prosocial behavior is voluntary acts beneficial to society, organization, and coworkers [50]. Such voluntary behaviors do not take place from external coercion or sense of duties, but one’s own moral and ethical values and consideration of others. Thus, as prosocial behaviors stem from one’s own thought and control as the autonomy aspect of psychological well-being [60], individuals who are engaged in prosocial behaviors are likely to have a higher level of psychological well-being than those who are not. Another behavioral aspect of individuals engaged in prosocial behaviors leading to a higher psychological well-being is positive relations with others, which contributes to building smooth interpersonal relation prosocial behaviors due to mutual relations of sympathy and trust. Furthermore, desirable prosocial behaviors can be regarded as a universally shared value arising from personal values, not from responsibilities or duties [66], resulting in a belief. Accordingly, such values and beliefs will have positive effects on realizing ultimate individual goals of life. To conclude, prosocial behaviors are closely associated with self-acceptance, positive relations with others, autonomy, purpose in life, and personal growth, which have positive effects on psychological well-being. Drawing on the above discussions, the following hypothesis is proposed:

**Hypothesis** **2b.** *Team members’ prosocial behaviors will have a positive effect on psychological well-being.*

### 2.3. Mediating Effect of the Team’s Value Congruence Environment and Team Members’ Prosocial Behaviors

The relationship between the leader’s perception of CSR and team members’ psychological well-being can explain more aspects through the mediation of the team’s value congruence environment or team members’ prosocial behaviors.

As a desirable corporate activity, CSR activities can lead to official corporate activity recognition, which can be more appropriately spread throughout the organization through close interactions between team leaders and team members. This will enhance the team environment and give rise to an important motivation for value congruence, thus exerting a positive effect on team members’ psychological well-being. In this relationship, value congruence will serve as an important path leading to enhanced psychological well-being through self-acceptance and positive relations with others established by harmoniously matching individual and organizational values [43] and reducing conflicts.

**Hypothesis** **3a.** *Team’s value congruence environment will mediate the relationship between leader’s perception of CSR perception and team members’ psychological well-being.*

Moreover, a team leader plays the role of the corporate spokesperson or messenger of officially pursued corporate values or goals in the relationship between the leader (once the CSR activities are perceived) and team members. According to social learning theory [67], team members will likely imitate the team leader by concentrating on his/her attitude and behavior in this process, especially when the team leader is trusted by the team members [68]. Consequently, the team members will have a positive perception of CSR activities, which are the company’s prosocial activities, and pursue isomorphism based on the perception of CSR. As a result, individual team members will take pride in the organization to which they belong and be more willing to commit themselves to prosocial behaviors which are beneficial to coworkers and the organization. As prosocial behaviors greatly enhance job satisfaction and provide motivation as devoted activities [69], team members’ psychological well-being is expected to rise. The following hypothesis is thus proposed:

**Hypothesis** **3b.** *Team members’ prosocial behaviors will mediate the relationship between leader’s CSR perception and team members’ psychological well-being*.

**Figure 1:**
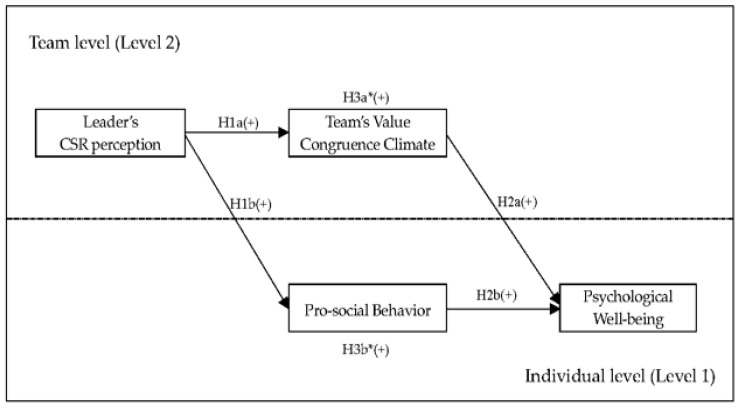
Study model. * Mediation effect; CSR = Corporate Social Responsibility; (+) = Positive relationship between variables.

Figure 1 is a schematic representation of the proposed study model.

## 3. Method

### 3.1. Sample and Procedure

The main target of this study was employees in various industries. More specifically, eight manufacturing companies (34.8%), 12 service companies (52%), and three financial companies (13%) were surveyed. As we strived to increase the reliability of empirical findings and the survey response rate, we contacted possible firms from different industries by using a human network. We then discussed whether the firms were suitable for our study in terms of importance of CSR activity and team role for their business. During this process, we reviewed not only the general status of CSR activities but also the key details of their personnel and the structure and characteristics of the team in the firms. We chose the members of the teams from the main task categories, including human resource, procurement, marketing, production, research and development, and others. The survey was conducted for about a month. Because this research analyses the levels of individuals and teams, we performed preliminary investigation for selecting proper companies. Then, we made an appointment for survey with manager of companies after explaining the academic purpose and delivering brief information of this research. The questionnaire was distributed to the participating teams after acquiring the permission from the companies. More specifically, we put the survey questionnaires in an envelope according to the number of team members and distributed it to the team representatives. The questionnaire was directed to be answered anonymously. For team-based analysis, an item that distinguishes team leaders and team members was included among the questions. Copies of the answered questionnaires were sealed and collected in an envelope for each team. By ensuring the confidentiality of the respondents, we could have more correct answers and reduced mistakes for team- based analysis. We sent questionnaires to 386 participants in 78 teams. After we excluding incomplete questionnaires and the teams less than six months old, 334 completed questionnaires (response rate 86.5%) in 69 qualified teams (response rate 88.4%) were finally used in our empirical analysis.

All data were used in the analysis. The results of the ANOVA showed no significant difference between industry and functional areas with main variables. All data were therefore used for final analysis. The minimum number of members of final 69 teams was 3 and the maximum was 11. The average number of members was 5.12 (SD = 0.50). The demographic characteristics were as follows. Gender distribution was 64.1% and 35.9% for men and women, respectively. Regarding age, the 30s were the largest group with 35.9%, followed by 27.2% in their 40s, 19.2% in their 20s, 15.6% in their 50s and 2.1% in their 60s or older. Regarding education, the group of university graduates was the highest with 55.4%, followed by 18.3% for college graduates, 17.1% for high school graduates or lower, 8.1% for masters and 1.2% for Ph.D. The average working period in the current team was 5.38 years (SD = 6.31) ranging from 1 to 30 years. By position of respondents, 38.6% were workers, 21.6% were deputy managers, 18.9% were managers, and 21.0% were department heads. By type of work, human resources accounted for 48.8%, procurement 7.5%, marketing 15.3%, production 9.9%, Research and Development 9.0%, and others 9.6%.

### 3.2. Measures

Surveys were written in Korean. Following Brislin’s [70] translation–back–translation procedure, two bilinguals in English and Korean performed two-way translations to ensure equivalency of meaning. Unless otherwise noted, every item was rated on a 5-point scale ranging from 1 (strongly disagree) to 5 (strongly agree).

#### 3.2.1. Leader’s CSR Perception

The degree of recognition and agreement of team leader about CSR was measured. We used 20 items consisting of six components (Economic, Legal, Ethical, Discretionary) used by Ryff and Keyes [60]. Sample items were “Our business has a procedure in place to respond to every customer complaint”, “All our products meet legal standards”, “We are recognized as a trustworthy company”, and “Our business supports local sports and cultural activities”. Cronbach’s alpha was 0.94 in this study.

#### 3.2.2. Value Congruence Environment

We used the three items that Cable and Judge [43] developed and Cable and DeRue [71] used. The three items were as follows; “The things that I value in life are very similar to the things that my organization values”, “My personal values match my organization’s values and culture”, and “My organization’s values and culture provide a good fit with the things that I value in life”. Leaders and individual employees’ responses were averaged out to calculate the mean score for a group-level construct of Value Congruence environment. Cronbach’s α of the summative scale was 0.91.

#### 3.2.3. Pro-Social Behaviors

Cooperative behavior among colleagues and additional behaviors other than defined roles and regulations in the job and defined as pro-social behavior to act voluntarily regardless of compensation. We used 15 items Podsakoff et al. [49] and Organ [50] developed and Bettencourt and Brown [72] reconstructed. It was divided into three areas of action (cooperation behavior, role-prescribed behavior, and extra-role behavior), each using five. Sample items were “Helps other employees who have heavy workloads”, “Does not take extra breaks”, and “Attends functions that are not required, but help the company image”. Cronbach’s α of the summative scale was 0.92.

#### 3.2.4. Psychological Well-Being

We used 19 items consisting of six components (Autonomy, Environmental Mastery, Personal Growth, Positive Relations With Others, Purpose in Life, and Self-Acceptance) developed by Ryff and Keyes [60]. Sample items were “Possesses a positive attitude toward the self”, “Has warm, satisfying, trusting relationships with others”, “Able to resist social pressures to think and act in certain ways”, “has a sense of mastery and competence in managing the environment”, “Has goals in life and a sense of directedness”, and “Has a feeling of continued development”. The response format was a five-point scale ranging from 1 (strongly disagree) to 5 (strongly agree). Cronbach’s alpha was 0.91 in this study.

#### 3.2.5. Control Variables

We controlled gender, age, and education level in pro-social behaviors and psychological well-being of employees. Team size and average team tenure were included at the team-level to partial out their potential effects on team and member performance.

## 4. Results

### 4.1. Descriptive Statistics and Correlations

Table 1 provides the means, standard deviations, reliabilities, correlations and rwg of the measures and variables used in the study. The statistics in the upper portion of the table pertain to the individual level of analysis. The data in the lower portion pertain to the correlations among team-level variables.

### 4.2. Confirmatory Factor Analysis

We conducted confirmatory factor analyses for the measures rated by team members and their leaders. In the analysis, the leader’s CSR perception was excluded because it was a single response directly assessed by the team leader, and the hypothesized model was measured for the measures (Leader’s CSR perception, Value congruence environment, Pro-social behaviors, Psychological well-being). These tests were performed at the individual level because individual ratings are likely to produce higher correlations among the study measures than averaged scores; thus, these individual-level tests are more conservative [73]. As a result, the three-factor model (χ2(df) = 749.22(313), CFI = 0.92, TLI = 0.91, RMR = 0.03, RMSEA = 0.06, NFI = 0.87, GFI = 0.86, AGFI = 0.83, IFI = 0.92, SRMR = 0.06) showed better model fit than the two-factor model (Value congruence climate, Pro-social behaviors and Psychological well-being; χ2(df) = 2043.38(323), CFI = 0.70, TLI = 0.67, RMR = 0.06, RMSEA= 0.12, NFI = 0.66, GFI = 0.55, AGFI = 0.47, IFI = 0.70, SRMR = 0.10) and the one-factor model (χ2(df) = 2532.67(324), CFI = 0.62, TLI = 0.58, RMR = 0.06, RMSEA= 0.14, NFI = 0.59, GFI = 0.52, AGFI = 0.44, IFI = 0.62, SRMR = 0.11). The χ2(chi square) test also showed that the three-factor model is superior to the other alternative models.

We further examined the discriminant validity of value congruence climate measures by comparing interrater agreement and reliability indices (rwg and ICCs). If the value of rwg(j), the confidence coefficient of the group, is more than 0.70, it can be justified to use the lower level data as group level data, in the case of ICC (1), the values of 0.05 to 0.20 are typical, and a level of 0.30 or better is a very good level [74]. In the case of ICC (2), the value of 0.50 to 0.70 can be partially accepted, and if it is 0.70 or higher, it can be evaluated as a good level [75]. The value of ICC (2) in the analysis result (rwg = 0.87, ICC(1) = 0.25, ICC(2) = 0.62, F = 2.66; *p* < 0.001) is slightly less than 0.70, but it can be partially accepted [74,76,77]. The F-test also showed a significant value, indicating that the value-congruence environment can be analyzed as a group variable. These results support the validity of the group variable measures of the multilevel analysis.

### 4.3. Hypothesis Tests

Given the multilevel nature of the study data, we used the Scientific Software International Hierarchical Linear Modeling (HLM) 7.01 program for data analysis [78,79,80]. We also performed multiple regression analyses to test the cross-level mediation hypotheses [81,82]. Table 2, Table 3 and Table 4 summarizes the results from hierarchical linear modeling (HLM) analyses. Our hypotheses imply that the significant variance in team member creativity can be explained at both team and individual levels. To test our hypotheses, we first had to ensure that significant team variance in PW existed. Otherwise, there was no point in moving to the team level and conducting further cross-level analyses. The basic model in Table 3 shows the information on the within-group variance and the between-group variance. As a result of analysis, the variable of individual level (σ2 = 0.23, *p* < 0.001) cannot be explained in the total variance of dependency variable, and the part that can be explained by the variable of group level (τ = 0.07, *p* < 0.001) was 24.1%. These results show that multilevel analysis is meaningful.

The hypotheses H1a and H1b predicted that CSR is positively associated with the team VCC and PB of team members. Table 2 shows the direct effects of leader CSR on team VCC and PB of team members. As shown in Table 2, the coefficient for on team VCC and team members PB was positive and significant (γ = 0.46, *p* < 0.001; γ = 0.39, *p* < 0.001). Therefore, H1a and H1b were supported.

The hypotheses H2a and H2b predicted that team VCC and team members PB were positively associated with the PW. Both H2a and H2b predicted that the VCC of the group level variable and the PB of the team member, which is the individual level variable, will have a positive effect on the dependent variable PW. The results of the analysis are shown in Table 3. Team VCC and team members PB showed significant results (γ = 0.43, *p* < 0.001; γ = 0.48, *p* < 0.001) for PW, respectively. Model 3 is the result of applying both individual level and group level variables and control variables. Group-level explanatory power (0.05 − 0.02 = 0.03) was higher than that of model 2. These results supported Hypothesis 2a and Hypothesis 2b.

Finally, we tested the mediator effect. Hypothesis H4a predicted that team VCC mediates the relationship between CSR and PW, and H4b predicted that team members PB mediate the relationship between CSR and PW. We used the criteria established by Baron and Kenny [81] to test interventions, and based on their suggestions, we followed three conditions to test the mediating role of team VCC and team members PB. First, CSR must be related to team VCC and team members PB; second, CSR must be related to team members PW; third, when controlling team VCC and team members PB as the mediating variable, the relationship between CSR as the independent variable and PW as the dependent variable must be much smaller than it is when CSR is the sole predictor. Thus, we tested H3a and H3b by examining the impact of CSR when team VCC and team members PB were entered into the model. Results in Table 2, Model 2 and Model 3, confirmed that CSR positively affected VCC and PB (γ = 0.46, *p* < 0.001; γ = 0.39, *p* < 0.001). Both Table 3 Model 2, and Table 4 Model 1, show that CSR positively affected PW (γ = 0.33, *p* < 0.001). In addition, CSR and parameters (VCC, PB), which are independent variables, were added together. Model 2 of Table 4 showed a significant positive result (γ = 0.43, *p* < 0.001) in relation to VCC and PW. Model 2 of Table 3 showed positive results (γ = 0.48, *p* < 0.001) in the relationship between PB and PW. And the CSR coefficient was smaller when it was the only predictive variable in relation to PW. The analyses showed that VCC and PB partially mediated the relationship between CSR and PW.

## 5. Discussion

### 5.1. Theoretical Contributions

Although CSR activities are directed towards both internal and external stakeholders, little research interest has been directed towards internal stakeholders. Therefore, this study focused on the effect of CSR activities on leader and employees, who are internal stakeholders. The study results confirmed the importance of the leader’s perception of CSR for improving employee’s psychological well-being through the mediation of group- and individual-level value congruence environment. These results support an important mechanism by which CSR activities help form the leader’s perception of CSR and enhance the team members’ psychological well-being, which is beneficial to organizational development [33,34,35,83,84]. This also demonstrates that the leader’s perception of CSR influences the team’s value congruence environment and plays a key role in improving employees’ prosocial behaviors.

As theoretical implications of this study, three aspects may be presented. First, whereas most CSR-related studies have revolved around external stakeholders [6,14,15,16], with little research devoted to the positive effect of CSR activities on internal stakeholders, this study sought to overcome this limitation by focusing on team leader and team members, who are internal stakeholders.

Second, whereas many studies on psychological well-being have been conducted from a health or sports study perspective [61,85,86,87], this study analyzed the leader–member relationship from the aspect of corporate organizational efficiency, using CSR activities, which is a corporate-level environment, as an important parameter. By considering the parameters conducive to the accomplishments of both company and staff, this study expanded the research on CSR activities and psychological well-being.

Third, this study identified the organization-level value congruence environment and team member’ prosocial behaviors as important parameters for the leader’s perception of CSR to exert a positive effect on team members’ psychological well-being. Specifically, multilevel analysis was applied to the process by which the leader’s perception of CSR activities yields positive outcomes at the individual and organizational levels.

### 5.2. Managerial Implications

As for the practical implications of this study, four aspects may be presented. First, this study confirmed the importance of a leader’s perception of CSR activities. Organizational members have limited information sources for the company’s CSR activities compared with the leader, inevitably resulting in limited positive effect of CSR activities. If team members are provided with enough information through the leader possessing a higher CSR perception level, the positive effect of the CSR activities on team members will increase. A company will have to leverage this role of a leader at the organizational level by ensuring that sufficient information on CSR activities is provided to the leader.

Second, considering that the attitude and behaviors of the leader provided with information on CSR activities yield significant results in employees’ value congruence environment and prosocial behaviors, there is a need to reflect this leader role in suitable leadership programs so that leaders can carry out their messenger role in an efficient manner. Third, considering that the promotion of team-level value congruence environment and prosocial behaviors help enhance the psychological well-being of team members (employees) and that enhanced psychological well-being has a positive effect on organizational development [88,89], active corporate intervention for creating value congruence environment and motivations for prosocial behaviors will have to be developed.

Finally, this study confirmed the importance of the leader’s role in improving team members’ psychological well-being which is favorable for organizational and individual development. Consequently, the importance of the leader role in CSR-related information collection and transmission to the team members needs to be recognized and necessary competence be cultivated.

### 5.3. Limitations and Future Research

Despite the contributions of this study, it has some limitations. They are presented along with the related future research directions.

First, our data was used cross-sectional data based on a point in time. In addition, the measurement of all variables used in this study was based on the subjective perception of participants. Despite our efforts to minimize the common method bias, we suggest that future research will have to increase reliability by diversifying the response source and measurement timing and applying a longitudinal study design to increase the causality of our findings. Second, this study did not consider the leader’s personal characteristics as a variable when transmitting his/her perception of CSR to the team members. The leader’s perception of CSR will have to be considered along with various individual level personality variables when analyzing outcomes. Third, the study results have the problem of generalizability because the data used are limited to employees working in teams of Korean companies. Therefore, there is a need to apply a research design considering multiple countries, business sectors, and occupation types.

## 6. Conclusions

Using a multilevel analysis model capable of simultaneous validation of team- and individual-level factors, we empirically investigated how a leader’s perception of corporate social responsibility influences team members’ psychological well-being. The findings of our study confirmed a positive effect of a leader’s CSR perception on team members’ psychological well-being and demonstrated the significant mediating roles of the value congruence environment and prosocial behavior among the team members in the relationship between them. Despite the limitations of the study discussed previously, this finding has important implications for organizations that need to improve team members’ psychological well-being which is key source of sustainable development of team and organization.

## Figures and Tables

**Table 1:** Descriptive statistics and correlations.

**(a) Individual (Level 1) Variables**		**Mean**	**SD**	**1**	**2**	**3**	**4**	**5**	**6**	**7**	**8**
1.	Gender	1.35	0.48	-							
2.	Age	2.45	1.03	−2.21 **	-						
3.	Education	2.58	0.90	−0.19	−0.20 **	-					
4.	Tenure	8.84	8.43	−0.31 **	0.58 **	−0.06	-				
5.	Position	2.22	1.16	−0.32 **	0.47 **	0.23 **	0.50 **	-			
6.	Job characteristic	2.00	0.37	−0.03	0.14 **	−0.18 **	0.00	−0.11 *	-		
7.	Prosocial behaviors	3.66	0.55	−0.15 **	0.17 **	0.24 **	0.21 **	0.28 **	0.06	(0.92)	
8.	Psychological well-being	3.68	0.55	−0.03	0.00	0.19 **	0.10	0.07	−0.01	0.55 **	(0.91)
**(b) Team (Level 2) Variables**		**Mean**	**SD**	**ICC1**	**ICC2**	**1**	**2**	**3**			
1.	Team size	4.82	1.62								
2.	Team tenure	5.18	4.18			0.14					
3.	Leader’s CSR perception	3.77	0.55			0.17	−0.12	(0.94)			
4.	Value congruence climate	3.52	0.49	0.25	0.62	0.05	−0.26 *	0.54 ***	(0.91/0.87)		

(a) *n* = 334 for level 1 variables; (b) *n* = 69 for level 2 variables. Values in parentheses in the upper portion and the first number in parentheses in the lower portion are alpha coefficients. The second numbers in the lower part’s parentheses are average interrater reliability (r_wg_’s). * *p* < 0.05; ** *p* < 0.01; *** *p* < 0.001, Two-tailed tests; SD = standard division; ICC(1) = Interclass Correlation Coefficients assessing the inter-respondent reliability; ICC(2) = Interclass Correlation Coefficients assessing the mean reliability of a group.

**Table 2:** Hierarchical linear model predicting: Direct effects.

Variables	Prosocial Behaviors (PB)		Value Congruence Climate (VCC)
	Model 1	Model 2	Model 3
Fixed effect	Estimates	Estimates	Estimates
Individual Level			
Intercept	3.68 ***	3.68 ***	
Gender		−0.02	
Age		0.11	
Education		0.10	
Group Level			
Intercept			1.86 ***
Team size		−0.04 *	0.00
Team tenure		−0.00	−0.02 **
Leader’s CSR perception (CSR)		0.39 ***	0.46 ***
Random effect	Variancecomponent	Variancecomponent	Variancecomponent
Group-level variance(*τ*)	0.3043 ***	0.0515 ***	0.0515 ***
Individual-level variance(*σ*^2^)	0.4675	0.2020	0.2020
Deviance	516.96	494.29	494.29
χ^2^		143.37	143.37

*n* = 334 for level 1 variables and 69 for level 2 variables. * *p* < 0.1; ** *p* < 0.05; *** *p* < 0.001; CSR = Corporate Social Responsibility.

**Table 3:** Hierarchical linear model predicting psychological well-being (PW): Direct and mediating effects (PB).

Variables	Psychological Well-being (PW)			
	Null Model	Model 1	Model 2	Model 3
Fixed effect	Estimates	Estimates	Estimates	Estimates
Individual Level				
Intercept	3.69 ***	3.69 ***	2.58 ***	1.77 ***
Gender		−0.08	−0.08	−0.08
Age		−0.04	−0.04	−0.04
Education		−0.00	−0.00	−0.00
Prosocial behaviors (PB)		0.48 ***	0.48 ***	0.48 ***
Group Level				
Team size			−0.02	−0.02 **
Team tenure			−0.00	0.00
Leader’s CSR perception (CSR)			0.33 ***	0.13 *
Value congruence climate (VCC)				0.43 ***
Random effect	Variance component	Variance component	Variance component	Variance component
Group-level variance(*τ*)	0.0755 ***	0.0861 ***	0.0567 ***	0.0245 ***
Individual-level variance(*σ*^2^)	0.2377	0.1906	0.1901	0.1910
Deviance	532.73	487.43	484.61	460.69
χ^2^		212.3	156.64	103.75

*n* = 334 for level 1 variables and 69 for level 2 variables. * *p* < 0.1; ** *p* < 0.05; *** *p* < 0.001.

**Table 4:** Mediating effect of value congruence climate (Group Level).

Variables	Psychological Well-being (PW)		
	Null Model	Model 1	Model 2
Fixed effect	Estimates	Estimates	Estimates
Intercept	3.69 ***	2.59 ***	1.77 ***
Team size		−0.02 **	−0.02
Team tenure		−0.00	0.00
Leader’s CSR perception (CSR)		0.33 ***	0.12 *
Value congruence climate (VCC)			0.43 ***
Random effect	Variance component	Variance component	Variance component
Individual-level variance (*σ*^2^)	0.2377	0.2369	0.2379
Group-level variance (*τ*)	0.0755 ***	0.0469 ***	0.0145 ***
Change in variance (∆*τ*)		0.0286	0.0324
Proportion of explained variance		37.8%	42.9%

*n* = 334 for level 1 variables and 69 for level 2 variables. * *p* < 0.1; ** *p* < 0.05; *** *p* < 0.001; CSR = Corporate Social Responsibility.
